# Sliding Ferroelectricity Driven Spin‐Layertronics in Altermagnetic Multilayers

**DOI:** 10.1002/advs.76050

**Published:** 2026-06-11

**Authors:** Rui Peng, Guangxu Su, Yangyang Fan, Jiaan Li, Fanxin Liu, Yee Sin Ang

**Affiliations:** ^1^ School of Physics Zhejiang University of Technology Hangzhou China; ^2^ Science, Mathematics and Technology Cluster Singapore University of Technology and Design Singapore Singapore

**Keywords:** altermagnetism, first‐principles calculations, layer degree of freedom, nonvolatile switching, sliding ferroelectricity

## Abstract

The synergy of ferroicity with altermagnetism offers a novel platform for designing multifunctional altermagnetic‐spintronic device technology. In this work, we propose a mechanism to achieve nonvolatile electrical manipulation of spin and layer degrees of freedom in an altermagnetic bilayer via sliding ferroelectricity. Using first‐principles calculations, we show that an interlayer translation can induce a switchable out‐of‐plane ferroelectric polarization in bilayer CuF_2_, which directly couples to and reverses the *d*‐wave altermagnetic spin splitting. Notably, the altermangetic spin splitting is layer‐locked, the sliding ferroelectricity‐driven switching thus embodying a nonvolatile spin‐layertronics functionality that couples spin‐polarized transport and layer degree of freedom in a single platform. We show that in quadrilayer CuF_2_, four polarization states are identified which may offer multi‐state logic device applications. These findings establish sliding ferroelectricity as a versatile tool for designing voltage‐controlled, high‐speed, and energy‐efficient spin‐layertronic devices based on altermagnets.

## Graphical abstract

Integrating sliding ferroelectricity with altermagnetism enables nonvolatile electrical control of spin and layer degrees of freedom. In bilayer CuF_2_, interlayer translation reverses layer‐locked spin‐split bands, establishing a multifunctional “spin‐layertronic” platform. This synergy facilitates energy‐efficient, multi‐state logic applications, offering a versatile route for designing high‐speed, voltage‐controlled altermagnetic spintronic devices based on van der Waals materials.

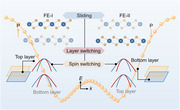

## 1 Introduction

Altermagnetism represents an emerging frontier in collinear magnetism, uniquely combining antiferromagnetic (AFM) symmetry with a momentum‐dependent spin splitting reminiscent of ferromagnets [1, 2, 3, 4, 5]. This novel class exhibits zero net magnetization, ensuring robustness against external fields and compatibility with high‐speed operations, while simultaneously hosting a non‐relativistic spin‐split electronic structure that is crucial for spin manipulation [6, 7, 8, 9, 10, 11, 12, 13, 14, 15, 16, 17, 18, 19, 20, 21, 22, 23, 24, 25, 26, 27, 28]. Such a dichotomy unlocks a wealth of emergent phenomena—including the generation of non‐collinear spin currents [1, 6], anomalous Hall effects [4, 10], and substantial tunneling magnetoresistance [7]—which collectively position altermagnets as a highly promising platform for next‐generation spintronics. The recent experimental confirmation of various altermagnetic candidates has further amplified their potential for ultra‐compact device integration [29, 30, 31, 32, 33, 34]. However, a central challenge persists: achieving efficient, purely electrical control over the altermagnetic order and its associated spin polarization remains essential for their seamless integration with modern electronics.

The pursuit of electrical control in altermagnets motivates their integration with ferroic orders, leading to the emerging class of multiferroic altermagnets [35, 36, 37, 38, 39, 40, 41, 42, 43, 44, 45]. One prominent example is the altermagnetoelectric effect, where ferroelectric (FE) polarization couples directly to the altermagnetic spin texture, allowing electrical switching of the magnetic order [35, 36, 37, 38, 39, 40, 41, 42]. This success invites the exploration of other ferroic couplings, such as with ferroelasticity, to achieve mechanical or strain‐based control [43, 44, 45]. A particularly promising yet distinct avenue is the integration of altermagnetism with sliding ferroelectricy—a phenomenon unique to van der Waals multilayers, in which a lateral interlayer translation induces interfacial charge redistribution and generates a switchable out‐of‐plane polarization [46, 47, 48, 49, 50, 51, 52, 53, 54, 55, 56, 57, 58, 59]. While major efforts have been focused on harnessing sliding ferroelectricy to manipulate unconventional magnetism in bilayer heterostructures [60, 61, 62], how the layer degree of freedom (DOF)—inherent in a multilayered system—couple cooperatively with both altermagnetic and sliding‐FE orders to tunable multifunctional behavior remains unexplored thus far. Critically, the simultaneous manipulation of the layer DOF and altermagnetism via sliding ferroelectricity shall substantially broaden the physics and application potential of multiferroic altermagnets.

**FIGURE 1:**
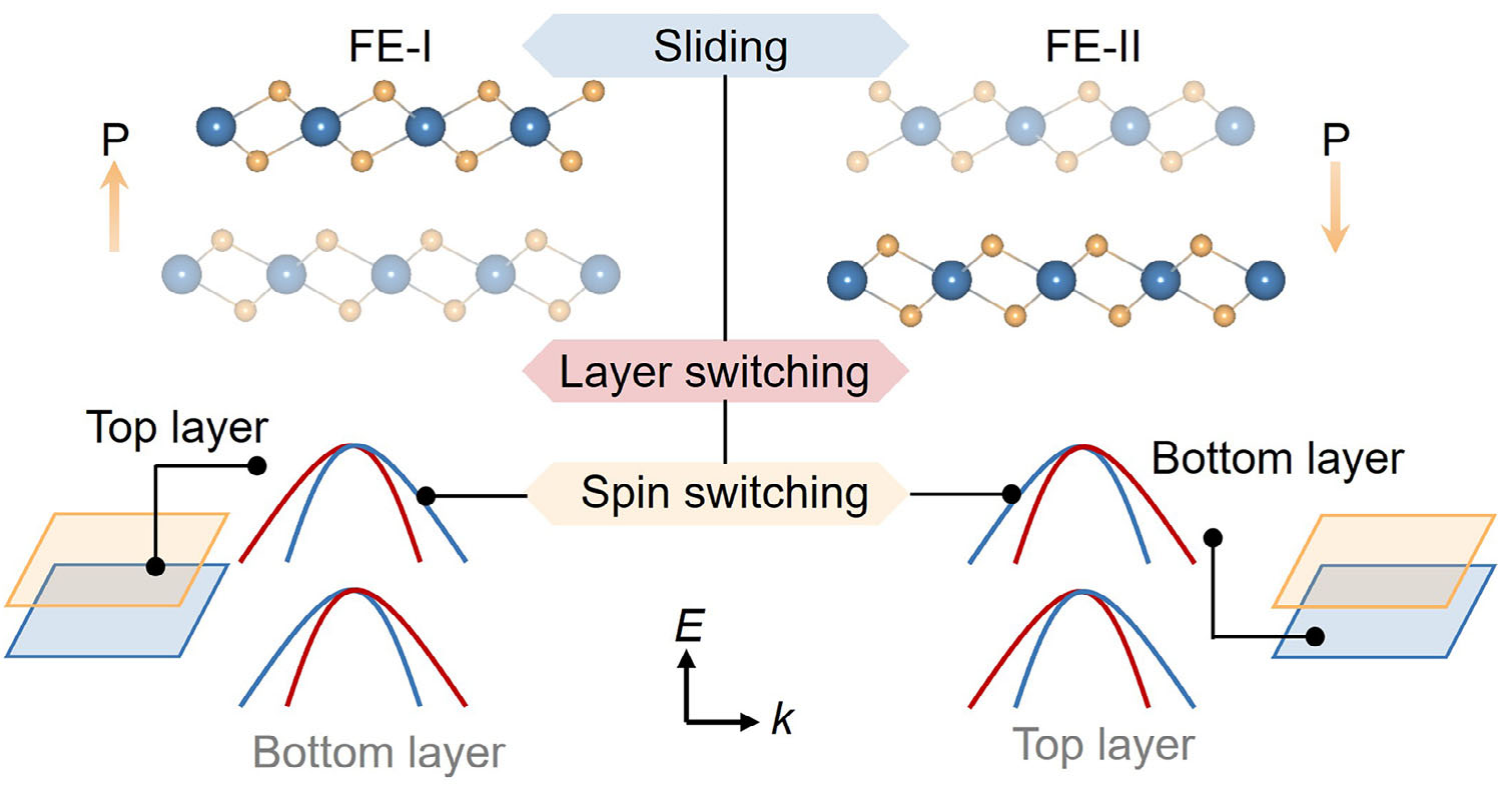
Concept of sliding‐ferroelectricity driven spin‐layertronic in an altermagnetic bilayer. Upon switching of the FE states (FE‐I and FE‐II), the built‐in electric polarization is reversed. The reversal of the FE polarization causes the altermagnetic spin‐split bands to energetically shift in the opposite direction for the two sub‐monolayers, thus leading to altermagnetic spin switching.

In this work, we propose the concept of nonvolatile switchable spin‐layertronics in two‐dimensional (2D) multiferroic altermagnet by integrating sliding ferroelectricity with altermagnetism and layer DOF in a bilayer setup. Using monolayer CuF_2_—a known altermagnet exfoliable from its bulk phase [63, 64]—as a building block, we show that bilayer CuF_2_ in specific stacking configurations exhibits intrinsic sliding ferroelectricity with two stable FE states, denoted FE‐I and FE‐II, and sizable out‐of‐plane polarization (± 1.23 pC/m) that enables efficient electrical control. Notably, the system exhibits layer‐locked altermagnetic spin‐split bands, which are energetically shifted in the opposite fashion by the intrinsic out‐of‐plane FE polarization. Switching between FE‐I and FE‐II thus reverses the energetic shifting of these layer‐locked altermagnetic spin‐split bands, resulting in altermangetic spin reversal (see Figure 1). By extending to quadrilayer CuF_2_, we identify four inequivalent FE polarization states, in which the layer and the spin‐polarized current can be separately controlled via sliding ferroelectricity, thus offering a spin‐layertronic platform to achieve multistate device functionalities. Our findings reveal sliding‐ferroelectricity‐driven altermagnetic bilayer as a viable platform for electrically controllable, multi‐state spin‐layertronic route toward advanced memory and logic devices.

## 2 Results and Discussion

### 2.1 Sliding Ferroelectricicty in Bilayer CuF_2_

Among the commonly reported 2D altermagnetic materials, several prototypical structural motifs prevail, including Cr_2_Se_2_O [6, 7, 12], CrS [8, 17], Fe_2_MX_4_ (M═Mo, W; X═S, Se, Te) [18], penta‐MnS_2_ [26], RuF_4_ [9, 14], and CuF_2_ [45] (see Table S1). For the first two structural families, the stable bilayer stacking configurations lack polarity and therefore preclude the emergence of sliding ferroelectricity. In contrast, the remaining candidates exhibit stacking arrangements that are compatible with ferroelectricity. From the perspective of material realization, both CuF_2_ and RuF_4_ can be obtained via exfoliation, making them suitable for constructing few layer and multilayer van der Waals heterostructures. In this work, we select CuF_2_ as a prototypical system to investigate sliding ferroelectricity driven spin layertronics. To understand the origin of the polarity in its bilayer stacking configuration, it is instructive to compare its monolayer structure with those of another two similar structures with the MX_2_ (M = metal, X = nonmetal) stoichiometry. Figure S1 compares the crystal structures of monolayers 1T‐MoS_2_ (space group **P** $\bar{3}$ **m1**; No. 164), 1T’‐WTe_2_ (space group **P2_1_/m**; No. 11) and CuF_2_ (space group **P2_1_/c**; No. 14). In monolayer 1T‐MoS_2_, the Mo atom is octahedrally coordinated by six S atoms. In monolayer 1T’‐WTe_2_, the W atoms undergo spontaneous dimerization due to Peierls distortion, resulting in a distorted octahedral coordination. In contrast, the octahedral coordination in monolayer CuF_2_ is distorted in a different manner, driven by the Jahn–Teller effect. Recent studies demonstrated the out‐of‐plane switchable electric polarization in bilayer 1T’ systems [47, 48, 52, 53]. This polarization arises from uncompensated interlayer vertical charge transfer, which can be switched through interlayer sliding. Inspired by these findings, we stack monolayers CuF_2_ into a bilayer system and explore the potential of sliding ferroelectricity in bilayer CuF_2_.

**FIGURE 2:**
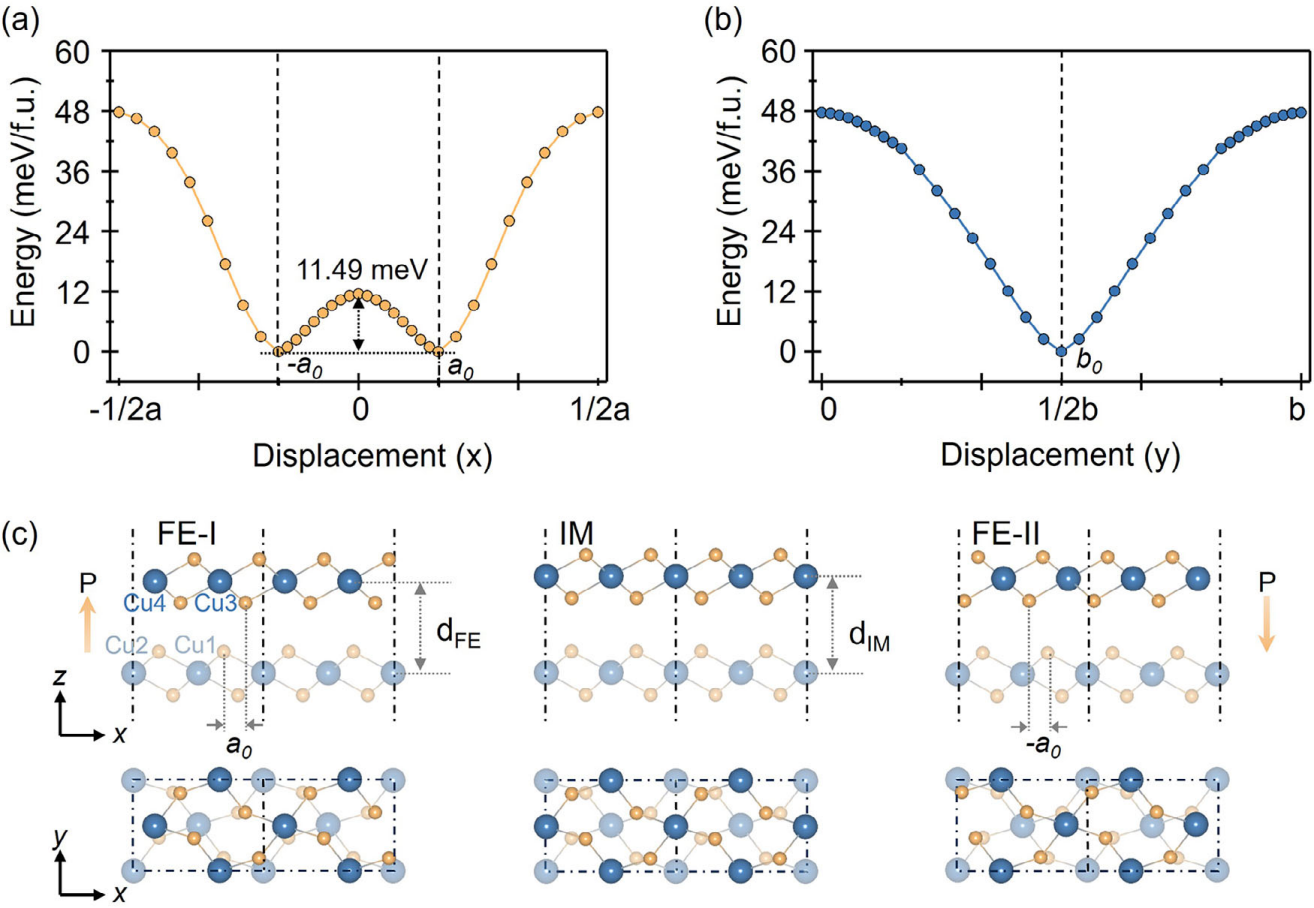
Energy profiles of bilayer CuF_2_ along (a) x‐ and (b) y‐directions. (c) Crystal structures of FE‐I, IM, and FE‐II states from side and top views.

The bilayer structure is constructed by stacking the top layer onto the bottom layer under the symmetry operation {m_z_|(t_x_, t_y_, d)}, where t_x_ and t_y_ represent the translation distances along the x‐ and y‐directions, respectively, and d denotes the interlayer distance. The energy landscapes of bilayer CuF_2_ as a function of t_x_ and t_y_ are presented in Figure 2a,b. As shown, the energy profile exhibits a double‐well potential along the x‐direction and a single‐well potential along the y‐direction. Energy minima occur at t_x_ = ±a_0_ and t_y_ = b_0_, where a_0_ = 1/6a and b_0_ = 1/2b (with a and b being the crystal constants of bilayer CuF_2_). Correspondingly, bilayers governed by {m_z_|(±a_0_, b_0_, d_FE_)} correspond to two equivalent FE states, labeled FE‐I and FE‐II, while the configuration under {m_z_|(0, b_0_, d_IM_)} represents an intermediate (IM) state, with d_FE_ and d_IM_ referring to the interlayer distances of the FE and IM states, respectively [see Figure 2c]. The switching barrier of bilayer CuF_2_ is 11.49 meV/f.u.

The space group of FE‐I (FE‐II) state is **Pc** (No. 7), which lacks inversion symmetry. The optimized lattice constants of FE‐I (FE‐II) state are a = 5.32 Å and b = 3.72 Å. The asymmetry in atomic positions between the top and bottom layers results in a corresponding shift of the charge centers, which directly gives rise to the spontaneous out‐of‐plane electric polarization in FE‐I (FE‐II) state. Using the Berry phase approach, the electric polarization is calculated to be +1.23 pC/m for FE‐I state and −1.23 pC/m for FE‐II state (see Figure S2). The presence of out‐of‐plane electric polarization is further corroborated by differential charge density analysis. As shown in the insets of Figure S2, a clear interlayer charge transfer in FE‐I state indicates an upward‐oriented electric polarization. Conversely, the direction of charge transfer is reversed in FE‐II state, confirming an oppositely oriented polarization. In contrast, the IM state crystallizes in the space group **Pca2_1_** (No. 29). The specific gliding symmetry present in the IM state suppresses the out‐of‐plane component of the electric polarization, leading to a net zero polarization along the stacking direction. To place the ferroelectric properties of bilayer CuF_2_ into context with other sliding ferroelectric materials, a detailed comparison is summarized in Table k. As shown in the Table S2, the electric polarization of the bilayer CuF_2_ (1.23 pC/m or 2.2*10^−3^ C/m^2^) is larger than that of 1T’‐WTe_2_ (5.1*10^−4^ C/m^2^) [47, 48] and remains competitive among known sliding ferroelectrics. While the switching barrier of bilayer CuF_2_ (11.49 meV/f.u.) is lower than that of conventional bulk ferroelectrics [50], it is comparable to or higher than that of bilayer h‐BN (∼4.5 meV/f.u.) [46] and bilayer 1T’‐WTe_2_ (∼0.15 meV/f.u.) [47], which are well‐known room‐temperature sliding ferroelectrics. The low collective barrier facilitates energy‐efficient switching, while the robust in‐plane chemical bonding prevents random thermal flipping of individual units due to the large activation area required for sliding. To further verify this, we performed ab initio molecular dynamics (AIMD) simulations at 300 K. As shown in Figure S3, the structure remains well within its initial stacking configuration over 5 ps without any sign of spontaneous switching or structural collapse, confirming that bilayer CuF_2_ is kinetically stable against thermal fluctuations at room temperature.

### 2.2 FE Reversal of Layer‐Locked Altermagnetic Spin‐Split Bands in Bilayer CuF_2_

In our previous work, monolayer CuF_2_ was identified as an altermagnet [45]. To determine the magnetic ground states of bilayer CuF_2_, we calculated the energy differences between C‐type and G‐type AFM orders for both the FE and IM states (see Figure S4). In FE‐I (FE‐II) state, the C‐type AFM order is found to be 0.78 meV per unit cell higher in energy than the G‐type AFM order, indicating that the G‐type AFM order is energetically favorable. The computed magnetic moments on the Cu sites of FE‐I (FE‐II) state are 0.625 (0.624), −0.625 (−0.624), −0.624 (−0.625), and 0.624 (0.625) µ_B_ for Cu1, Cu2, Cu3, and Cu4, respectively. Due to symmetry breaking, the Cu atoms in the top (bottom) layer exhibit slightly smaller magnetic moments than those in the bottom (top) layer, although the net magnetic moment remains zero. Similarly, in the IM state, the C‐type AFM order is 2.12 meV per unit cell higher than the G‐type AFM, confirming the G‐type order as the magnetic ground state.

**FIGURE 3:**
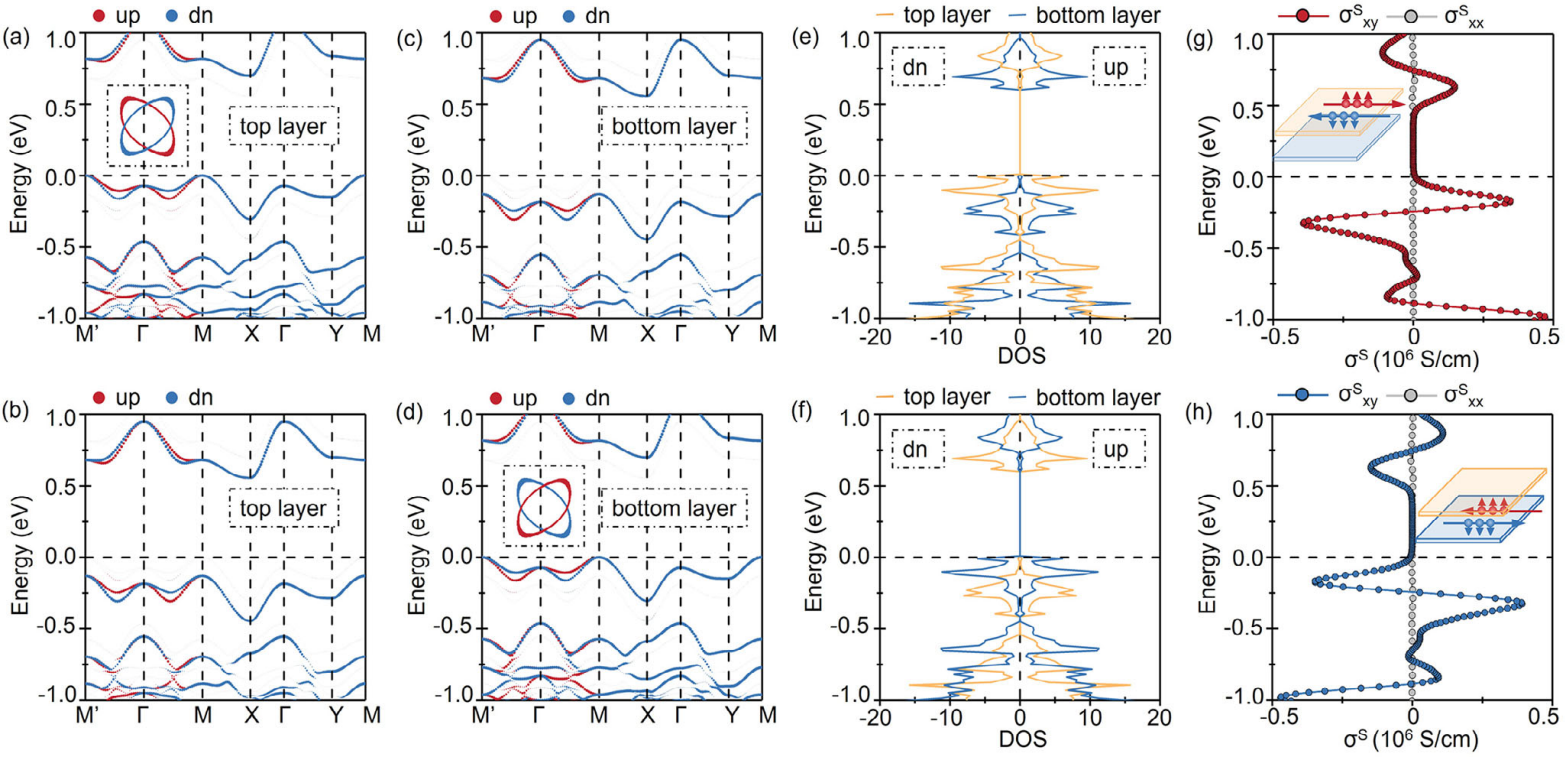
Layer‐resolved spin‐polarized band structures of (a) FE‐I and (b) FE‐II states with the contribution from the top layer. Inset in (a) is the isoenergy contours near the Fermi level of FE‐I state. Layer‐resolved spin‐polarized band structures of (c) FE‐I and (d) FE‐II states with the contribution from the bottom layer. Inset in (d) is the isoenergy contours near the Fermi level of FE‐II state. Density of states of (e) FE‐I and (f) FE‐II states. Fermi level is set to zero in (a) to (f). Longitudinal and transverse spin conductivities in (g) FE‐I and (h)FE‐II states. Insets are schematic diagrams of the layer‐polarized spin currents in FE‐I and FE‐II states.

In order to examine whether the synergistic switch of electric and spin/layer polarizations exists in this system, we plot the layer‐resolved spin‐polarized band structures and density of states for FE‐I and FE‐II states in Figure 3, and those of IM state in Figure S5. In the IM state, altermagnetic spin splitting is preserved within each layer, with opposite spin splitting between the two layers, forming a layer‐locked spin‐momentum coupling. The energy bands are layer‐degenerate due to the absence of out‐of‐plane electric polarization, which leads to spin degeneracy across the entire system. From a symmetry perspective, the out‐of‐plane glide symmetry, when combined with the global spinor rotation {m_z_ | 0 1/2 0}U, prohibits spin splitting. This is because the glide operation exchanges the two atoms with opposite spin—Cu1 and Cu3 (and similarly Cu2 and Cu4)—while leaving the 2D k‐space invariant, thereby enforcing spin degeneracy throughout the 2D Brillouin zone.

In contrast, the layer degeneracy is broken in FE‐I (FE‐II) state due to the presence of out‐of‐plane electric polarization. As shown in Figure 3a–f, the conduction band minimum (CBM) of FE‐I (FE‐II) state is mainly from the bottom (top) layer, while the valence band maximum (VBM) is domanited by the top (bottom) layer. The layer polarization energy, determined to be 0.13 eV (−0.13) eV for the FE‐I (FE‐II) state, is defined as the energy offset between the layer‐projected VBM of the two constituent layers. This results in a net altermagnetic spin splitting in the band structures of both FE‐I and FE‐II states, resembling that of monolayer CuF_2_. The spin‐up and spin‐down states remain degenerate along M–X–Γ–Y–M, while altermagnetic spin splitting emerges along M′–Γ–M. Furthermore, the spin‐momentum coupling pattern is opposite between FE‐I and FE‐II states. Upon FE switching, the spin polarization is simultaneously reversed, enabling nonvolatile control of the altermagnetic spin splitting. This spin reversal is also evident in the isoenergy contours of FE‐I and FE‐II states, further confirming the feasibility of manipulating altermagnetic spin splitting through FE polarization switching, as illustrated in the insets of Figure 3a,d.

### 2.3 Sliding Ferroelectricity Driven Spin‐Layertronics in Multilayer CuF_2_

The reversal of altermagnetic spin splitting leads to concomitant changes in other physical properties. In altermagnets, an anisotropic spin splitting allows an external in‐plane electric field to generate a spin current [1, 6]. The spin conductivity of bilayer CuF_2_ can be calculated using the Boltzmann transport equation. As illustrated in Figure 3g, the longitudinal spin conductivity in FE‐I state is zero, while a sizable signal appears in the transverse direction. Notably, upon FE switching, the conductivity reverses sign owing to the opposite spin‐momentum coupling patterns between FE‐I and FE‐II states, as shown in Figure 3h. These results demonstrate that FE polarization can effectively control spin currents in bilayer CuF_2_, enabling nonvolatile spin information encoding and electric‐field‐controllable spintronic device applications.

It is noteworthy that the layer polarization is accompanied by spin polarization, which in turn results in a layer‐polarized spin current. Specifically, in the FE‐I and FE‐II states, the spin current is predominantly localized in the top and bottom layers, respectively, thereby endowing the spin current with an additional layer DOF; see the insets in Figure 3g,h. Consequently, the layer‐spin polarization can alternatively be detected by examining the layer localization of the spin current. We describe a state exhibiting coexisting spin and layer polarization that is switchable via FE polarization as (P, S, L), where P = ± 1 corresponds to upward and downward electric polarization, S = ± 1 represents spin‐up and spin‐down currents, and L = ± 1 denotes the top and bottom layers. Accordingly, the FE‐I and FE‐II states can be expressed as (+1,+1,+1) and (−1,−1,−1), respectively, implying the synergistic regulation of spin current and layer occupation by ferroelectric switching.

**FIGURE 4:**
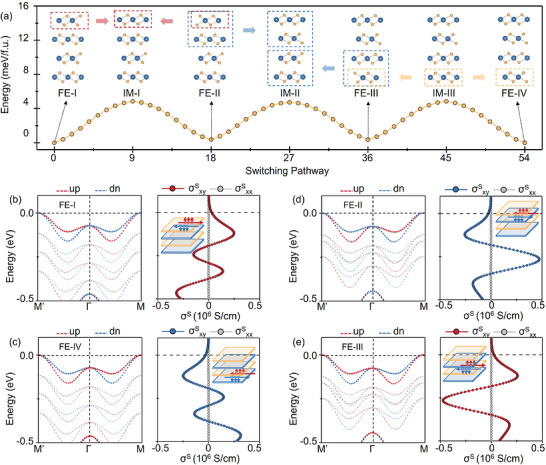
(a) Energy profiles for the ferroelectric sliding pathway (FE‐I — FE‐II — FE‐III — FE‐IV). Layer‐resolved spin‐polarized band structures, and longitudinal and transverse spin conductivities of (b) FE‐I, (c) FE‐IV, (d) FE‐II, and (e) FE‐III states. Fermi level is set to zero. Insets are schematic diagrams of the layer‐polarized spin currents in quadrilayer CuF_2_.

The interplay between electric, spin and layer polarization can be further extended to multilayer systems. For instance, in quadrilayer CuF_2_, interlayer sliding gives rise to four inequivalent polarization states: FE‐I, FE‐II, FE‐III and FE‐IV; see Figure 4a. The FE‐I state, characterized by consecutive AA stacking across two FE‐I configurations of bilayer CuF_2_, naturally exhibits upward electric polarization. Applying a glide operation to this state yields the FE‐IV state, which reverses the out‐of‐plane electric polarization direction. Similarly, the FE‐II state, formed by AA stacking across FE‐I and FE‐II configurations of bilayer CuF_2_, also shows upward electric polarization, and its glide counterpart FE‐III state reverses the electric polarization. Owing to their distinct stacking sequences, the absolute electric polarization values differ between the FE‐I/FE‐IV and FE‐II/FE‐III states. FE‐I/FE‐IV states exhibit a larger electric polarization of ±4.22 pC/m, whlie FE‐II/FE‐III states have an electric polarization of ±1.09 pC/m. To assess the experimental feasibility, we investigated the switching dynamics among these states. As illustrated in Figure 4a, a sequential, step‐wise sliding pathway (FE‐I — FE‐II — FE‐III — FE‐IV) is identified. At each switching stage, relative displacement occurs at only one specific van der Waals interface, while the layers on either side of the interface move as rigid blocks. Such an interface‐by‐interface transition is kinetically favored over collective multi‐layer sliding, as it significantly reduces the energy cost by overcoming only one interfacial potential barrier at a time. The calculated energy barriers are consistently low (6.06 meV/f.u. for FE‐I — FE‐II and FE‐III — FE‐IV, and 5.44 meV/f.u. for FE‐II — FE‐III), confirming that these transitions are experimentally feasible under external stimuli, thereby enabling robust multi‐state logic operations.

The band structures of these four states, as shown in Figure 4b–e, reveal a more sophisticated layer‐dependent band‐edge localization compared to the bilayer system. For FE‐I and FE‐IV states, the VBM is predominantly localized in the two outer layers, whereas for FE‐II and FE‐III, the VBM shifts to the two inner layers. This dramatic shift provides a tunable layer DOF to spin currents through specific atomic planes. To provide direct theoretical evidence for the multi‐state spin‐layertronic functionality, we calculated the spin conductivity for all four states. Figure 4b–e shows that the spin conductivity not only undergoes sign reversals upon polarization switching but also exhibits variations in magnitude across different stacking sequences. For instance, the transition from FE‐I to FE‐II involves a redistribution of the spin current from the outer to the inner layers, accompanied by a distinct change in the spin conductivity profile.

To extend the (P, S, L) framework to the quadrilayer system, we characterize the four distinct states as a quaternary logic system: (+2,+1,+2) for FE‐I, (+1,−1,−1) for FE‐II, (−1,+1,+1) for FE‐III, and (−2,−1,−2) for FE‐IV. For the electric polarization P, ±2 and ±1 denote the high‐polarization (±4.22 pC/m) and low‐polarization (±1.45 pC/m) states, respectively. For the layer index L, ±2 corresponds to the spin current being localized in the outer layers, while ±1 represents the inner layers, and the parity of the index determines the specific layer occupation. Remarkably, these three degrees of freedom exhibit a strict synergy governed by a unified relation: sgn(S) = sgn(L) = sgn(P)·(‐1)^|P|+N/2^, where N is the total number of layers. This formula encapsulates the fundamental coupling and synchronous switching of the spin current and layer occupation as the ferroelectric polarization evolves. Furthermore, this synergistic relationship can be generalized to other even‐numbered multilayer systems (N≥2), providing a general design principle for electric‐field‐controllable spin‐layertronic leapfrog devices based on sliding ferroelectricity.

Table S3 offers a comprehensive comparison between the CuF_2_ multilayer and other representative multiferroic spin‐layertronic platforms based on sliding ferroelectricity, highlighting several fundamental distinctions that set our work apart. Specifically, in terms of magnetic configuration, CuF_2_ exhibits a G‐type antiferromagnetic order where every nearest‐neighbor spin is antiparallel. This differs from the A‐type or C‐type orders typically reported in systems such as MnBi_2_Te_4_ [54] or AgF_2_ [61], making CuF_2_ a rare example of a sliding ferroelectric candidate with a fully compensated G‐type magnetic structure, which is advantageous for minimizing stray fields and enabling high‐frequency spin dynamics. Furthermore, while prior studies have primarily focused on charge‐based signals such as the layer‐polarized anomalous Hall effect (AHE) or anomalous valley Hall effect (AVHE) [54, 55, 59, 61], the CuF_2_ system directly generates a layer‐polarized pure spin current upon electric switching. This feature provides an alternative avenue for low‐power spintronic devices where power dissipation from charge currents is largely eliminated. Finally, moving beyond the bilayer‐centric focus of most existing literature, our work provides a systematic investigation into both bilayer and quadrilayer configurations. By deriving a unified scaling rule—sgn(S) = sgn(L) = sgn(P)·(‐1)^|P|+N/2^—for even‐layered systems, we establish a predictive framework for generalizing these effects to thicker stacks, thereby establishing CuF_2_ multilayers as a versatile and unique platform for advanced spin‐layertronics.

## 3 Conclusion

In summary, we have proposed and theoretically validated a strategy to achieve nonvolatile FE‐switchable spin‐layertronics by integrating altermagnetism with sliding ferroelectricity in multilayer heterostructures. We demonstrated that interlayer sliding induces out‐of‐plane FE polarization in bilayer CuF_2_ which directly couples to and reverses the layer‐locked d‐wave altermagnetic spin‐splitting texture. Transport calculations revealed that the corresponding layer‐locked spin conductivities can be ferroelectrically encoded, highlighting the potential of sliding FE altermagnets as nonvolatile nanomechanical spin‐layer switches. Extending to quadrilayer CuF_2_, we identify four inequivalent polarization states, thus revealing a material platform for electrically controllable, multi‐state spin‐layer logic and memory devices. Our work not only addresses the key challenge of multiferroic switching in altermagnets, but also establishes FE control of spin‐layertronic that enriches the device application landscape of altermangets.

## 4 Methods

First‐principles calculations are performed based on density functional theory (DFT) [65] as implemented in Vienna ab initio simulation package (VASP) [66]. Exchange‐correlation interaction is described by the Perdew‐Burke‐Ernzerhof (PBE) parametrization of generalized gradient approximation (GGA) [67]. Structures are relaxed until the force on each atom is less than 0.01 eV/Å. The cutoff energy and electronic iteration convergence criterion are set to 500 eV and 10^−5^ eV, respectively. To sample the 2D Brillouin zone, a Monkhorst–Pack (MP) k‐grid mess [68] of 5 × 9 × 1 is used for bilayer CuF_2_. To avoid the interaction between adjacent layers, a vacuum space of 20 Å is added. FE switching barrier is obtained by nudged elastic band (NEB) method [69]. Berry phase approach is employed to evaluate the out‐of‐plane electric polarization [70]. The spin‐resolved transport properties are calculated using a housing‐made code, in which the electron energy and electron group velocity are evaluated from the Wannier‐based tight binding Hamiltonian [71]. To assess the influence of electron correlation on our main conclusions, we performed DFT+U calculations with U = 0, 1, 2, and 3 eV. As shown in Table S4, increasing U leads to a moderate increase in the magnetic moment (from 0.625 to 0.725 µ_B_) and the ferroelectric polarization (from 1.23 to 1.34 pC/m). The altermagnetic spin splitting remains significant (54–67 meV) throughout this range. Importantly, the qualitative symmetry‐protected altermagnetic features and the sliding ferroelectric mechanism are robust against the choice of U. This demonstrates that the PBE functional provides a reliable description of the core physical phenomena in this system.

## Author Contributions

R.P. and Y.S.A. conceived and designed the project. R.P. performed the calculations and analysis. R.P. and Y.S.A. wrote the manuscript with inputs from all authors. F.X.L. reviewed and analysed the results. All the authors discussed the contents and prepared the manuscript.

## Conflicts of Interest

The authors declare no conflicts of interest.

## Supporting information




**Supporting File**: advs76050‐sup‐0001‐SuppMat.pdf.

## Data Availability

The data that support the findings of this study are available from the corresponding author upon reasonable request.

## References

[1] R. González‐Hernández , L. Šmejkal , K. Výborný , et al., “Efficient Electrical Spin Splitter Based on Nonrelativistic Collinear Antiferromagnetism,” Physical Review Letters 126 (2021): 127701. 33834809 10.1103/PhysRevLett.126.127701

[2] L. Šmejkal , J. Sinova , and T. Jungwirth , “Emerging Research Landscape of Altermagnetism,” Physical Review X 12 (2022): 040501.

[3] L. Šmejkal , J. Sinova , and T. Jungwirth , “Beyond Conventional Ferromagnetism and Antiferromagnetism: a Phase with Nonrelativistic Spin and Crystal Rotation Symmetry,” Physical Review X 12 (2022): 031042.

[4] L. Šmejkal , A. H. MacDonald , J. Sinova , S. Nakatsuji , and T. Jungwirth , “Anomalous Hall Antiferromagnets,” Nature Reviews Materials 7 (2022): 482.

[5] L. Bai , W. Feng , S. Liu , L. Šmejkal , Y. Mokrousov , and Y. Yao , “Altermagnetism: Exploring New Frontiers in Magnetism and Spintronics,” Advanced Functional Materials 34 (2024): 2409327.

[6] H. Y. Ma , M. Hu , N. Li , et al., “Multifunctional Antiferromagnetic Materials with Giant Piezomagnetism and Noncollinear Spin Current,” Nature Communications 12 (2021): 2846. 10.1038/s41467-021-23127-7 PMC8121910 33990597

[7] Q. Cui , Y. Zhu , X. Yao , P. Cui , and H. Yang , “Giant Spin‐Hall and Tunneling Magnetoresistance Effects Based on a Two‐Dimensional Nonrelativistic Antiferromagnetic Metal,” Physical Review B 108 (2023): 024410.

[8] X. Chen , D. Wang , L. Li , and B. Sanyal , “Giant Spin‐Splitting and Tunable Spin‐Momentum Locked Transport in Room Temperature Collinear Antiferromagnetic Semimetallic CrO Monolayer,” Applied Physics Letters 123 (2023): 022402.

[9] J. Sødequist and T. Olsena , “Two‐Dimensional Altermagnets from High Throughput Computational Screening: Symmetry Requirements, Chiral Magnons, and Spin‐Orbit Effects,” Applied Physics Letters 124 (2024): 182409.

[10] H. Jin , Z. Tan , Z. Gong , and J. Wang , “Anomalous Hall Effect in Two‐Dimensional Vanadium Tetrahalogen with Altermagnetic Phase,” Physical Review B 110 (2024): 155125.

[11] R.‐W. Zhang , C. Cui , R. Li , et al., “Predictable Gate‐Field Control of Spin in Altermagnets with Spin‐Layer Coupling,” Physical Review Letters 133 (2024): 056401. 39159119 10.1103/PhysRevLett.133.056401

[12] J. Gong , Y. Wang , Y. Han , et al., “Hidden Real Topology and Unusual Magnetoelectric Responses in Two‐Dimensional Antiferromagnets,” Advanced Materials 36 (2024): 2402232. 10.1002/adma.202402232 38684179

[13] S. Zeng and Y. Zhao , “Description of Two‐Dimensional Altermagnetism: Categorization Using Spin Group Theory,” Physical Review B 110 (2024): 054406.

[14] M. Milivojević , M. Orozović , S. Picozzi , M. Gmitra , and S. Stavrić , “Interplay of Altermagnetism and Weak Ferromagnetism in Two‐Dimensional RuF_4_ ,” 2D Materials 11 (2024): 035025.

[15] S. Zeng and Y. Zhao , “Bilayer Stacking A‐Type Altermagnet: a General Approach to Generating Two‐Dimensional Altermagnetism,” Physical Review B 110 (2024): 174410.

[16] Z. Jin , Z. Zeng , Y. Cao , and P. Yan , “Skyrmion Hall Effect in Altermagnets,” Physical Review Letters 133 (2024): 196701. 39576911 10.1103/PhysRevLett.133.196701

[17] R. Peng , J. Yang , L. Hu , et al., “All‐Electrical Layer‐Spintronics in Altermagnetic Bilayers,” Materials Horizons 12 (2025): 2197–2207. 40067761 10.1039/d4mh01509f

[18] Y. Li , Y. Zhang , X. Lu , et al., “Ferrovalley Physics in Stacked Bilayer Altermagnetic Systems,” Nano Letters 25 (2025): 6032–6039. 40192027 10.1021/acs.nanolett.4c06037

[19] X. Chen , J. Ren , J. Li , Y. Liu , and Q. Liu , “Unconventional Magnons in Collinear Magnets Dictated by Spin Space Groups,” Nature 640 (2025): 349–354. 40074910 10.1038/s41586-025-08715-7 PMC11981943

[20] Y. Che , Y. Chen , X. Liu , H. Lv , X. Wu , and J. Yang , “Inverse Design of 2D Altermagnetic Metal–Organic Framework Monolayers from Hückel Theory of Nonbonding Molecular Orbitals,” JACS Au 5 (2025): 381–387. 39886576 10.1021/jacsau.4c01150 PMC11775709

[21] D. Bezzerga , I. Khan , and J. Hong , “High Performance Room Temperature Multiferroic Properties of w ‐MnSe Altermagnet,” Advanced Functional Materials 35 (2025): 2505813.

[22] D. Jeong , S.‐H. Kang , and Y.‐K. Kwon , “Magnetic and Crystal Symmetry Control on Spin Hall Conductivity in Altermagnets,” Advanced Science 13 (2025): 15002. 10.1002/advs.202515002 PMC12955886 41387290

[23] C. Song , H. Bai , Z. Zhou , et al., “Altermagnets as a New Class of Functional Materials,” Nature Reviews Materials 10 (2025): 473–485.

[24] M. Hu , X. Cheng , Z. Huang , and J. Liu , “Catalog of C‐Paired Spin‐Momentum Locking in Antiferromagnetic Systems,” Physical Review X 15 (2025): 021083.

[25] F. Zhang , X. Cheng , Z. Yin , et al., “Crystal‐Symmetry‐Paired Spin–Valley Locking in a Layered Room‐Temperature Metallic Altermagnet Candidate,” Nature Physics 21 (2025): 760–767.

[26] J. Wang , X. Yang , Z. Yang , et al., “Pentagonal 2D Altermagnets: Material Screening and Altermagnetic Tunneling Junction Device Application,” Advanced Functional Materials 36 (2025): 2505145.

[27] D. Wang , A. K. Ghosh , Y. Tao , F. Ma , and C. Song , “Emerging of Anomalous Higher‐Order Topological Phases in Altermagnet/Topological Insulator Heterostructure by Floquet Engineering,” Advanced Science 13 (2026): 22203. 10.1002/advs.202522203 PMC13045236 41618838

[28] K. Dou , Z. He , W. Du , Y. Dai , B. Huang , and Y. Ma , “Altermagnetic Skyrmions in 2D lattices exhibiting anisotropic Skyrmion Hall effect,” Physical Review B arXiv: 2505.05154 113 (2026): 104402.

[29] M. Leiviskä , J. Rial , A. Bad'ura , et al., “Anisotropy of the Anomalous Hall Effect in Thin Films of the Altermagnet Candidate Mn_5_Si_3_ ,” Physical Review B 109 (2024): 224430.

[30] L. Han , X. Fu , R. Peng , et al., “Electrical 180° Switching of Néel Vector in Spin‐Splitting Antiferromagnet,” Science Advances 10 (2024): adn0479. 10.1126/sciadv.adn0479 PMC10816707 38277463

[31] J. Krempaský , L. Smejkal , S. W. D'Souza , et al., “Altermagnetic Lifting of Kramers Spin Degeneracy,” Nature 626 (2024): 517–522. 38356066 10.1038/s41586-023-06907-7 PMC10866710

[32] S. Lee , S. Lee , S. Jung , et al., “Broken Kramers Degeneracy in Altermagnetic MnTe,” Physical Review Letters 132 (2024): 036702. 38307068 10.1103/PhysRevLett.132.036702

[33] M. Hajlaoui , S. W. D'Souza , L. Smejkal , et al., “Temperature Dependence of Relativistic Valence Band Splitting Induced by an Altermagnetic Phase Transition,” Advanced Materials 36 (2024): 2314076. 10.1002/adma.202314076 38619144

[34] Z. Zhou , X. Cheng , M. Hu , et al., “Manipulation of the Altermagnetic Order in CrSb via Crystal Symmetry,” Nature 638 (2025): 645–650. 39939768 10.1038/s41586-024-08436-3

[35] M. Gu , Y. Liu , H. Zhu , et al., “Ferroelectric Switchable Altermagnetism,” Physical Review Letters 134 (2025): 106802. 40153660 10.1103/PhysRevLett.134.106802

[36] X. Duan , J. Zhang , Z. Zhu , et al., “Antiferroelectric Altermagnets: Antiferroelectricity Alters Magnets,” Physical Review Letters 134 (2025): 106801. 40153648 10.1103/PhysRevLett.134.106801

[37] W. Sun , C. Yang , W. Wang , et al., “Proposing Altermagnetic‐Ferroelectric Type‐III Multiferroics with Robust Magnetoelectric Coupling,” Advanced Materials 37 (2025): 2502575. 40211656 10.1002/adma.202502575 PMC12232234

[38] Z. Zhu , X. Duan , J. Zhang , B. Hao , I. Žutić , and T. Zhou , “Two‐Dimensional Ferroelectric Altermagnets: from Model to Material Realization,” Nano Letters 25 (2025): 9456–9462. 40411465 10.1021/acs.nanolett.5c02121

[39] Z. Zhu , Y. Liu , X. Duan , et al., “Emergent Multiferroic Altermagnets and Spin Control via Noncollinear Molecular Polarization,” Science China Physics, Mechanics & Astronomy 68 (2025): 127562.

[40] Y. Yang , “Giant Bulk Spin Photovoltaic Effect in the Two‐Dimensional Altermagnetic Multiferroics VOX_2_ ,” Physical Review B 112 (2025): 104427.

[41] L. Šmejkal , “Altermagnetic multiferroics and altermagnetoelectric effect,” arXiv: 2411.19928v1.

[42] W. Guo , J. Xu , Y. Yang , H. Wang , and H. Zhang , “Altermagnetic type‐II multiferroics with Néel‐order‐locked electric polarization,” Nat Commun arXiv: 2505.01964v2 (2025), 10.1038/s41467-026-73750-5. PMC13389291 42203803

[43] N. Ding , H. Ye , S. Wang , and S. Dong , “Ferroelastically Tunable Altermagnets,” Physical Review B 112 (2025): L220410.

[44] Y. Huang , C. Hua , R. Xu , J. Liu , Y. Zheng , and Y. Lu , “Spin Inversion Enforced by Crystal Symmetry in Ferroelastic Altermagnets,” Physical Review Letters 135 (2025): 266701. 41557391 10.1103/jql2-b4c4

[45] R. Peng , S. Fang , P. Ho , et al., “Ferroelastic Altermagnetism,” npj Quantum Materials 11 (2026): 5.

[46] L. Li and M. Wu , “Binary Compound Bilayer and Multilayer with Vertical Polarizations: Two‐Dimensional Ferroelectrics, Multiferroics, and Nanogenerators,” ACS Nano 11 (2017): 6382–6388. 28602074 10.1021/acsnano.7b02756

[47] Q. Yang , M. Wu , and J. Li , “Origin of Two‐Dimensional Vertical Ferroelectricity in WTe_2_ Bilayer and Multilayer,” The Journal of Physical Chemistry Letters 9 (2018): 7160–7164. 30540485 10.1021/acs.jpclett.8b03654

[48] Z. Fei , W. Zhao , T. A. Palomaki , et al., “Ferroelectric Switching of a Two‐Dimensional Metal,” Nature 560 (2018): 336–339. 30038286 10.1038/s41586-018-0336-3

[49] M. V. Stern , Y. Waschitz , W. Cao , et al., “Interfacial Ferroelectricity by van der Waals Sliding,” Science 372 (2021): 1462. 10.1126/science.abe8177 34112727

[50] M. Wu and J. Li , “Sliding Ferroelectricity in 2D van der Waals Materials: Related Physics and Future Opportunities,” Proceedings of the National Academy of Sciences 118 (2021): 2115703118. 10.1073/pnas.2115703118 PMC8685923 34862304

[51] T. Zhang , Y. Liang , X. Xu , B. Huang , Y. Dai , and Y. Ma , “Ferroelastic‐Ferroelectric Multiferroics in a Bilayer Lattice,” Physical Review B 103 (2021): 165420.

[52] Y. Wan , T. Hu , X. Mao , et al., “Room‐Temperature Ferroelectricity in 1T′‐ReS_2_ Multilayers,” Physical Review Letters 128 (2022): 067601. 35213175 10.1103/PhysRevLett.128.067601

[53] T. Zhang , X. Xu , Y. Dai , B. Huang , and Y. Ma , “Intrinsic Ferromagnetic Triferroicity in Bilayer T′‐VTe_2_, Appl,” Physics Letters 120 (2022): 192903.

[54] R. Peng , T. Zhang , Z. He , et al., “Intrinsic Layer‐Polarized Anomalous Hall Effect in Bilayer MnBi_2_Te_4_ ,” Physical Review B 107 (2023): 085411.

[55] T. Zhang , X. Xu , B. Huang , Y. Dai , L. Kou , and Y. Ma , “Layer‐Polarized Anomalous Hall Effects in Valleytronic van der Waals Bilayers,” Materials Horizons 10 (2023): 483–490. 36196974 10.1039/d2mh00906d

[56] Z. Wang , Z. Gui , and L. Huang , “Sliding Ferroelectricity in Bilayer Honeycomb Structures: a First‐Principles Study,” Physical Review B 107 (2023): 035426.

[57] T. H. Yang , B.‐W. Liang , H.‐C. Hu , et al., “Ferroelectric Transistors Based on Shear‐Transformation‐Mediated Rhombohedral‐Stacked Molybdenum Disulfide,” Nature Electronics 7 (2024): 29–38.

[58] Y. Guo , R. Meng , X. Hu , et al., “Sliding Ferroelectricity in Kagome‐B_2_X_3_ (X = S, Se, Te) Bilayers,” Applied Physics Letters 124, 152901 (2024).

[59] T. Zhang , M. Wang , X. Xu , Y. Dai , and Y. Ma , “Gate‐Controllable Quadri‐Layertronics in a 2D Multiferroic Antiferromagnet,” Materials Horizons 12 (2025): 6919–6927. 40521942 10.1039/d5mh00242g

[60] W. Sun , W. Wang , C. Yang , et al., “Altermagnetism Induced by Sliding Ferroelectricity via Lattice Symmetry‐Mediated Magnetoelectric Coupling,” Nano Letters 24 (2024): 11179–11186. 39213606 10.1021/acs.nanolett.4c02248

[61] Y. Zhu , M. Gu , Y. Liu , et al., “Sliding Ferroelectric Control of Unconventional Magnetism in Stacked Bilayers,” Physical Review Letters 135 (2025): 056801. 40824793 10.1103/dmzg-ck2t

[62] Z. Guo , S. Qian , X. Zhou , W. Wang , Z. Cheng , and X. Wang , “Sliding Ferroelectric Metal with Ferrimagnetism,” Nature Communications 17 (2026): 549. 10.1038/s41467-025-67240-3 PMC12808114 41392132

[63] C. Billy and H. M. Haendler , “The Crystal Structure of Copper(II) Fluoride,” Journal of the American Chemical Society 79 (1957): 1049–1051.

[64] D. Jezierski and W. Grochala , “Polymorphism of Two‐Dimensional Antiferromagnets, AgF_2_ and CuF_2_ ,” Physical Review Materials 8 (2024): 034407.

[65] W. Kohn and L. J. Sham , “Self‐Consistent Equations Including Exchange and Correlation Effects,” Physical Review 140 (1965): A1133–A1138.

[66] G. Kresse and J. Furthmüller , “Efficient Iterative Schemes for Ab Initio Total‐Energy Calculations Using a Plane‐Wave Basis Set,” Physical Review B 54 (1996): 11169–11186. 10.1103/physrevb.54.11169 9984901

[67] J. P. Perdew , K. Burke , and M. Ernzerhof , “Generalized Gradient Approximation Made Simple,” Physical Review Letters 77 (1996): 3865–3868. 10062328 10.1103/PhysRevLett.77.3865

[68] H. J. Monkhorst and J. D. Pack , “Special Points for Brillouin‐Zone Integrations,” Physical Review B 13 (1976): 5188–5192.

[69] R. N. Barnett and U. Landman , “Born‐Oppenheimer Molecular‐Dynamics Simulations of Finite Systems: Structure and Dynamics of (H_2_O)_2_ ,” Physical Review B 48 (1993): 2081–2097. 10.1103/physrevb.48.2081 10008598

[70] R. D. King‐Smith and D. Vanderbilt , “Theory of Polarization of Crystalline Solids,” Physical Review B 47 (1993): 1651–1654. 10.1103/physrevb.47.1651 10006189

[71] A. A. Mostofi , J. R. Yates , G. Pizzi , et al., “An Updated Version of wannier90: a Tool for Obtaining Maximally‐Localised Wannier Functions,” Computer Physics Communications 185 (2014): 2309–2310.

