# Supplementary material for: Sliding Ferroelectricity Driven Spin‐Layertronics in Altermagnetic Multilayers
Source: Adv Sci (Weinh). 2026 Jun 11:e76050. Online ahead of print. doi: 10.1002/advs.76050 (PMC13336915; doi:10.1002/advs.76050)
Supplement: Supplementary file 1 — Supporting File: advs76050‐sup‐0001‐SuppMat.pdf. [file ADVS-9999-e76050-s001.pdf]

Supplementary Information

Sliding Ferroelectricity Driven Spin-Layertronics in Altermagnetic Multilayers

Rui Peng[1,*], Guangxu Su[1], Yangyang Fan[1], Jiaan Li[1], Fanxin Liu[1,†], Yee Sin Ang[2,‡]

[1] School of Physics, Zhejiang University of Technology, Hangzhou 310023, China

[2] Science, Mathematics and Technology Cluster, Singapore University of Technology and Design, Singapore 487372, Singapore.

Email: *pengrui@zjut.edu.cn, †liufanxin@zjut.edu.cn, ‡yeesin_ang@sutd.edu.sg

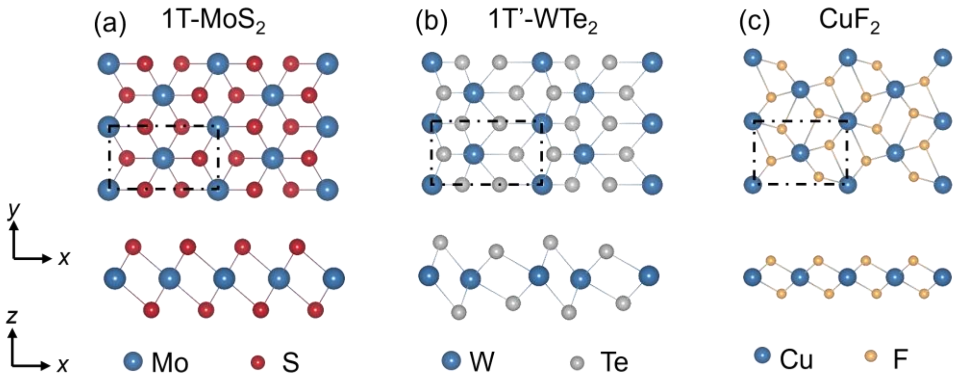

Fig.S1 Crystal structures of monolayers (a) 1T-MoS_2, (b) 1T'-WTe_2 and (c) CuF_2 from top and side views.

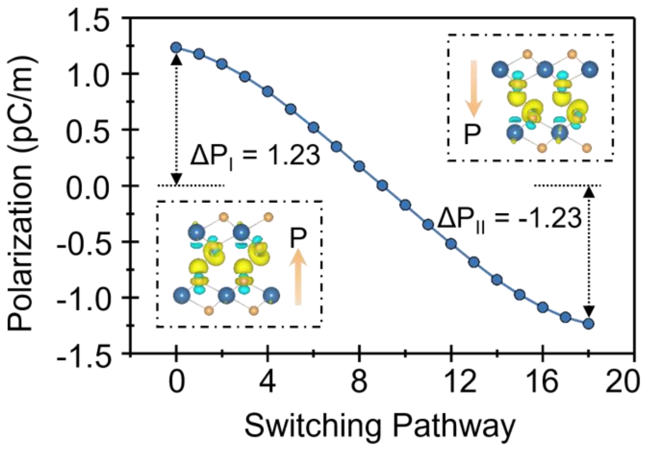

Fig. S2 Electric polarization in bilayer CuF_2 as a function of step number in NEB calculations. Insets show the differential charge density diagrams of FE-I and FE-II states with an

isosurface value of 0.002 e/Å³. Yellow and blue isosurfaces represent electron accumulation and depletion, respectively.

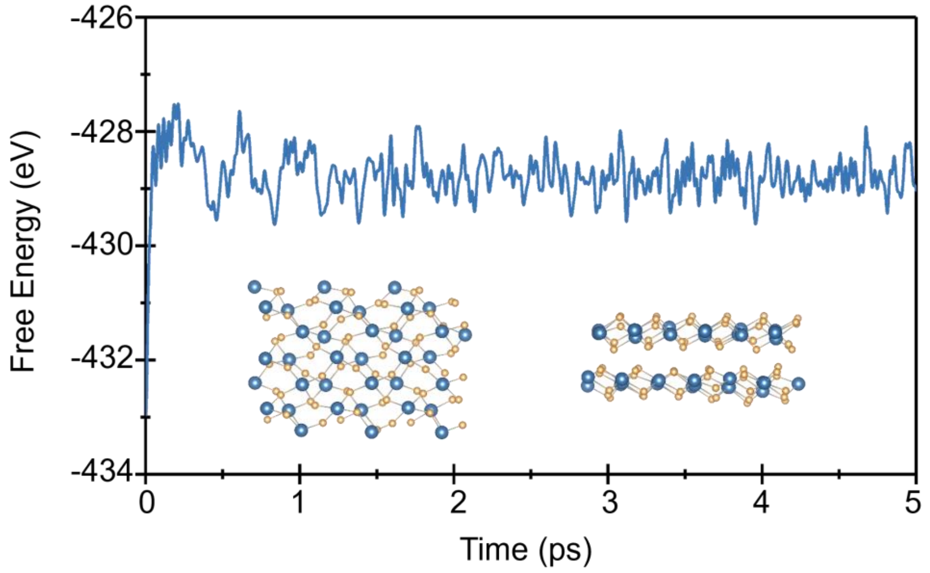

Fig.S3 Variations of the total energies at 300 K during AIMD simulations of monolayer CuF_2. Insets show the snapshots of the equilibrium structures.

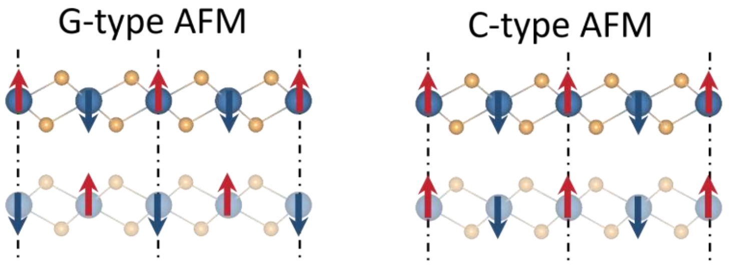

Fig. S4 G-type and C-type AFM configurations for bilayer CuF_2.

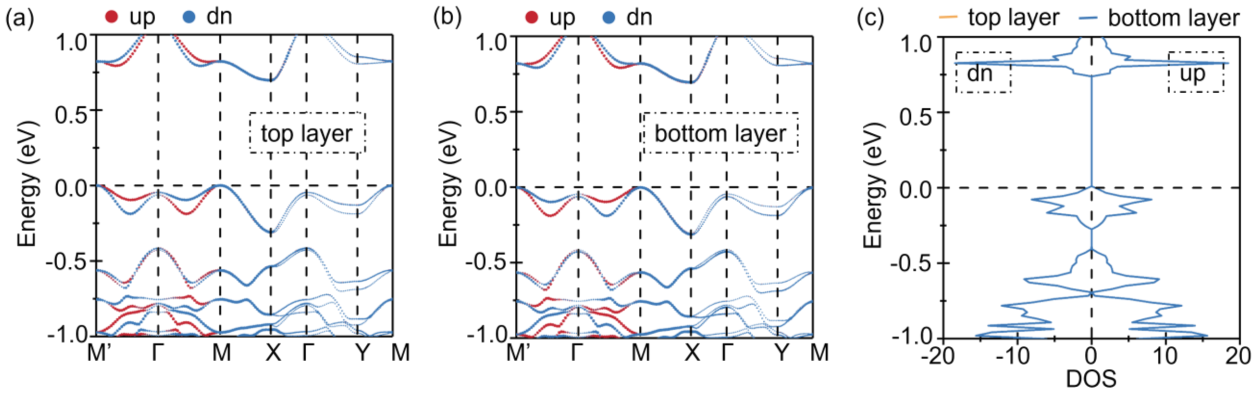

Fig. S5 Layer-resolved spin-polarized band structures of IM state with the contribution from (a) top and (b) bottom layers. (c) Density of states of IM state. Fermi level is set to zero.

Table S1 Comparison with other 2D altermagnetic candidates.

Material	Lattice	Space Group	Band Gap (eV)	Splitting (meV)	Potential Synthesis Method
Cr_2Se_2O [1]	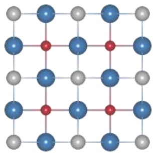	P4/mmm	1.12	1120	Synthetic
CrS [2]	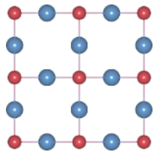	P4/mmm	0.3	300	Synthetic
Fe_2MX_4 (M = Mo, W; X = S, Se, Te) [3]	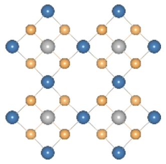	P-42m	0.4-1.01	-	Synthetic
Penta-MnS_2 [4]	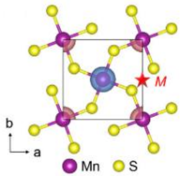	$P2_1/c$	0.79	109	Synthetic
RuF_4 [5]	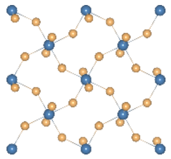	$P2_1/c$	0.83	163	Exfoliation
CuF_2 [6]	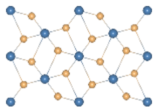	$P2_1/c$	0.69	98	Exfoliation

Table S2 Comparison of sliding ferroelectric parameters.

Materials	Electric Polarization	Switching Barrier (meV/f.u.)
h-BN bilayer [7]	2.08 pC/m	~4.5
WTe_2 bilayer [8]	$5.1*10^{-4}$ C/m^2	~0.15
ZrI_2 [9]	$2.1*10^{-4}$ C/m^2	1.6
VTe_2 [10]	$2.84*10^{-4}$	~10
CuF_2 bilayer (this work)	1.23 pC/m ($2.2*10^{-3}$ C/m^2)	11.49
CuF_2 quadrilayer (this work)	1.09-4.22 pC/m ($8.3*10^{-4}$-$3.2*10^{-3}$ C/m^2)	5.44-6.06

Table S3 Comparison of multiferroic spin-layertronic platforms.

Material	Multiferroic order	Effects	Number of Layer
$MnBi_2Te_4$ [11]	A-type AFM + FE	Layer-polarized AHE	2
VSi_2P_4 bilayer [12]	A-type AFM + FE	Layer-polarized AVHE	2
$OsCl_2$ quadrilayer [13]	A-type AFM + FE	Layer-polarized AVHE	4
AgF_2 bilayer [14]	C-type AFM +FE	Layer-polarized AHE	2
CuF_2 multilayer (this work)	G-type AFM+FE	Layer-polarized spin current	2+4

Table S4 Comparison of magnetic moment (μ_B), ferroelectric polarization (pC/m) and altermagnetic spin splitting (meV) among U = 0, 1, 2, and 3 eV.

U (eV)	Magnetic moment (μ_B)	Ferroelectric polarization (pC/m)	Altermagnetic spin splitting (meV)
0	0.624/0.625	1.23	54
1	0.666/0.667	1.31	67
2	0.697/0.698	1.34	64
3	0.724/0.725	1.34	59

References

1. Hidden real topology and unusual magnetoelectric responses in two-dimensional antiferromagnets, Adv. Mater. 36, 2402232 (2024).
2. R. Peng, J. Yang, L. Hu, W.-L. Ong, P. Ho, C. S. Lau, J. Liu, and Y. S. Ang, All-electrical layer-spintronics in altermagnetic bilayer, Mater. Horiz. 12, 2197 (2025).
3. Y. Li, Y. Zhang, X. Lu, Y. Shao, Z. Bao, J. Zheng, W. Tong, and C. Duan, Ferrovalley physics in stacked bilayer altermagnetic systems, Nano Lett. 25, 6032 (2025).
4. J. Wang, X. Yang, Z. Yang, J. Lu, P. Ho, W. Wang, Y. S. Ang, Z. Cheng, and S. Fang, Pentagonal 2D altermagnets: Material screening and altermagnetic tunneling junction device application, Adv. Funct. Mater. 2505145 (2025).
5. M. Milivojević, M. Orozović, S. Picozzi, M. Gmitra, and S. Stavrić, Interplay of altermagnetism and weak ferromagnetism in two-dimensional RuF_4, 2D Mater. 11, 035025 (2024).
6. R. Peng, S. Fang, P. Ho, F. Liu, T. Zhou, J. Liu, Y. S. Ang, Ferroelastic altermagnetism, npj Quantum Mater. 11, 5 (2026).

7. L. Li and M. Wu, Binary compound bilayer and multilayer with vertical polarizations: Two-dimensional ferroelectrics, multiferroics, and nanogenerators, ACS Nano 11, 6382 (2017).
8. Q. Yang, M. Wu, and J. Li, Origin of two-dimensional vertical ferroelectricity in WTe_2 bilayer and multilayer, J. Phys. Chem. Lett. 9, 7160 (2018).
9. R. Peng, T. Zhang, Z. He, Q. Wu, Y. Dai, B. Huang, and Y. Ma, Intrinsic layer-polarized anomalous Hall effect in bilayer $MnBi_2Te_4$, Phys. Rev. B 107, 085411 (2023).
10. T. Zhang, X. Xu, B. Huang, Y. Dai, L. Kou and Y. Ma, Layer-polarized anomalous Hall effects in valleytronic van der Waals bilayers, Mater. Horiz. 10, 483 (2023).
11. T. Zhang, M. Wang, X. Xu, Y. Dai and Y. Ma, Gate-controllable quadri-layertronics in a 2D multiferroic antiferromagnet, Mater. Horiz. 12, 6919 (2025).
12. Y. Zhu, M. Gu, Y. Liu, X. Chen, Y. Li, S. Du, and Q. Liu, Sliding ferroelectric control of unconventional magnetism in stacked bilayers, Phys. Rev. Lett. 135, 056801 (2025).
